# Genome Variation and Molecular Epidemiology of Salmonella enterica Serovar Typhimurium Pathovariants

**DOI:** 10.1128/IAI.00079-18

**Published:** 2018-07-23

**Authors:** Priscilla Branchu, Matt Bawn, Robert A. Kingsley

**Affiliations:** a Quadram Institute Bioscience, Norwich Research Park, Colney, Norwich, United Kingdom; b Earlham Institute, Norwich Research Park, Colney, Norwich, United Kingdom; Texas A&M University Health Science Center

**Keywords:** Salmonella, Typhimurium, epidemiology, genomics, host adaptation, phylogenetics, virulence

## Abstract

Salmonella enterica serovar Typhimurium is one of approximately 2,500 distinct serovars of the genus Salmonella but is exceptional in its wide distribution in the environment, livestock, and wild animals. *S*. Typhimurium causes a large proportion of nontyphoidal Salmonella (NTS) infections, accounting for a quarter of infections, second only to S. enterica serovar Enteritidis in incidence. *S*. Typhimurium was once considered the archetypal broad-host-range Salmonella serovar due to its wide distribution in livestock and wild animals, and much of what we know of the interaction of Salmonella with the host comes from research using a small number of laboratory strains of the serovar (LT2, SL1344, and ATCC 14028). But it has become clear that these strains do not reflect the genotypic or phenotypic diversity of *S*. Typhimurium. Here, we review the epidemiological record of *S*. Typhimurium and studies of the host-pathogen interactions of diverse strains of *S*. Typhimurium. We present the concept of distinct pathovariants of *S*. Typhimurium that exhibit diversity of host range, distribution in the environment, pathogenicity, and risk to food safety. We review recent evidence from whole-genome sequencing that has revealed the extent of genomic diversity of *S*. Typhimurium pathovariants, the genomic basis of differences in the level of risk to human and animal health, and the molecular epidemiology of prominent strains. An improved understanding of the impact of genome variation of bacterial pathogens on pathogen-host and pathogen-environment interactions has the potential to improve quantitative risk assessment and reveal how new pathogens evolve.

## Abstract (precis)

Salmonella enterica serovar Typhimurium is one of approximately 2,500 distinct serovars of the genus Salmonella but is exceptional in its wide distribution in the environment, livestock, and wild animals. *S*.

## INTRODUCTION

Salmonella remains a leading enteric bacterial pathogen in humans, domesticated animals, and wild animals worldwide. Infections in humans manifest as gastroenteritis, disseminated diseases (also called invasive nontyphoidal Salmonella disease) including bacteremia and enteric fever, or focal infections. The outcome of infection depends on the combination of the pathogen genotype, host species, and host immune status. The burden of disease caused by NTS in the human population is estimated to be up to 129.5 million cases each year, resulting in between 100,000 and 1 million deaths per year worldwide (1–4). Most infections result in a self-limiting gastroenteritis with a low mortality rate. The majority of mortality in recent years, up to 700,000 deaths per year, is from around 2 to 3 million cases of disseminated NTS, which primarily affects immunocompromised people in sub-Saharan Africa (5).

Salmonella serovars are distinguished by antigenic variation in long-chain lipopolysaccharides (LPS) and flagella and exhibit considerable variation in host range and outcome of infection (6). Two species comprise the genus, S. bongori and S. enterica. The latter is further subdivided into 2,500 serovars belonging to seven subspecies (I, II, IIIa, IIIb, IV, VI, and VII). All Salmonella strains are pathogens, due to the acquisition of major virulence factors early in the evolution of the genus, including a type III secretion system (T3SS) encoded on Salmonella pathogenicity island 1 (SPI-1) and, in the case of S. enterica, a second T3SS encoded on SPI-2 (7, 8). Salmonella is considered to be primarily a gastrointestinal pathogen, since this is a common feature of most serovars. However, host adaptation is often accompanied by a more severe disseminated disease; examples of such host adaptation are thought to arise by evolution from ancestors associated with gastroenteritis (6). Distinct host ranges and pathogenicities of Salmonella species, subspecies, and serovars are evident from epidemiological surveillance, clinical descriptions of disease in various hosts, and experimental infections. S. enterica and S. bongori are likely to differ in disease potential and severity, with the latter generally considered less virulent because it lacks the SPI-2-encoded type III secretion system, which is involved in enhanced intracellular replication (9, 10). Of eight subspecies comprising S. enterica, subspecies I (enterica) accounts for the vast majority of human infections, probably because of its widespread distribution in domesticated animals, while S. bongori and non-subspecies I serovars of S. enterica are generally associated with cold-blooded animals (11). Subspecies I serovars exhibit a general host adaptation to warm-blooded animals and specific host adaptation on a spectrum from broad to exquisitely host restricted (6, 12). Host adaptation refers to the ability to circulate in particular host species, and host-adapted strains may also be pathovariants due to altered pathogenicity (6). Evolution of subspecies I resulted in over 1,500 serovars, with a few evolving to become host adapted and, therefore, restricted to circulation in a single host species or closely related species. For example, S. enterica serovar Typhi and S. enterica serovar Gallinarum circulate in human and Galliformes (e.g., chicken) populations, respectively. Others exhibit a marked host preference and are associated with disseminated disease, such as S. enterica serovar Choleraesuis and S. enterica serovar Dublin, whose preferences are for pigs and cattle, respectively. The host ranges of most of the approximately 1,500 serovars of S. enterica subspecies I are poorly defined but are generally considered to be relatively broad. However, the distribution of Salmonella serovars in host species is far from homogenous in domesticated animals, suggesting a degree of host preference. Surveillance in the United Kingdom reveals distinct proportions of each serovar isolated from a different host species (13). Around 95% of isolates from each host species are accounted for by 6 to 11 serovars, with the remainder made up of many minority serovars (13). However, it is striking that in reports of the prevalence of Salmonella serovars in animal species, *S*. Typhimurium is the only serovar that appears as one of the top two serovars for virtually all host species (13, 14). For this reason, *S*. Typhimurium has been considered the prototypical broad-host-range serovar. However, the epidemiology of *S*. Typhimurium suggests a more complex mixture of pathovariants, some of which are highly host adapted. While some strains of *S*. Typhimurium do indeed have a broad host range, others exhibit a limited distribution within a single or related group of host species and may also exhibit distinct pathogenicities and risks to food safety (15–20).

## EPIDEMIOLOGY AND HOST RANGE OF *SALMONELLA* TYPHIMURIUM PATHOVARIANTS

**FIG 1:**
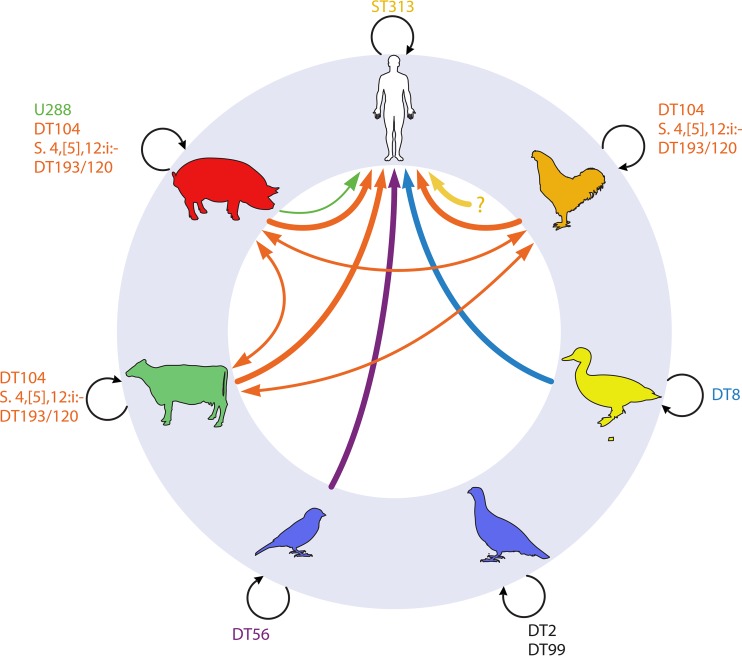
Host range and plausible transmission routes of major *S*. Typhimurium phage types in humans, pigs, cattle, wild avian species, ducks, and poultry. Dominant phage types are indicated in boldface, and minority phage types in italics. Arrow colors matched to each phage type indicate probable transmission routes based on the epidemiological record but may not include all possible routes. Line thickness indicates relative level of transmission, and the question mark a hypothetical route of transmission of ST313.

Subtyping of *S*. Typhimurium (antigenic formula 4,[5],12:i:1,2) for the purpose of outbreak detection has historically been achieved using phage typing in many countries worldwide, phage typing being a scheme based on differential levels of susceptibility to lysis by a panel of bacteriophages (21). The molecular basis of susceptibility is not known but is likely to be influenced by variations in LPS and surface proteins that are receptors for phages, modification and restriction systems, and superinfection exclusion systems encoded by prophages (22). National surveillance program data provide over half a century of epidemiological data about the host ranges and dynamics of *S*. Typhimurium epidemics caused by individual phage types (Table 1 and Fig. 1) (14, 15). Inferences of epidemiology and host range from phage type data must, however, be treated with caution because phage sensitivity does not provide direct information about the phylogenetic relationship of a strain. A particular phage type may in some cases be polyphyletic, and therefore, two isolates with the same phage type may potentially be nonclonal and exhibit distinct host ranges and pathogenicities. However, a temporally defined increase in the incidence of a particular phage type is used to detect outbreaks and, over longer time intervals, to define epidemics (21). It is important to note that there is no evidence that phage sensitivity itself has any direct impact on host range and/or pathogenesis, although this cannot be discounted entirely. Phage typing is, however, an inexpensive and convenient surrogate for the determination of phylogenetic groups. In comparison, multilocus sequence typing (MLST), a subgenomic scheme using sequences from seven alleles, does provide phylogenetic information, but this information is of relatively low resolution and therefore not commonly used for surveillance (23).

**TABLE 1:** Epidemiology and host ranges of Salmonella Typhimurium pathovariants

Phage type	Sequence type	Host range and epidemiology	Reference(s)
DT9	ST19	Broad host range (primarily cattle), MDR epidemic strain 1960 to 1975	21
DT204	ST19	Broad host range (primarily cattle), MDR epidemic strain 1970 to 1985	21, 106
DT104	ST19	Broad host range (primarily cattle), MDR pandemic strain 1990 to 2010	21, 64, 98
DT193/DT120^*a*^	ST34	Broad host range (primarily porcine), MDR pandemic 2005 to present	21, 59
U288	ST19	Porcine, MDR epidemic clone in pigs, 2000 to present, rare in humans	13, 107, 108
DT8	ST19	Ducks/geese	27–29
DT56var	ST568	Wild birds (passerines)	30, 67
DT2	ST98	Wild birds (pigeon), endemic in pigeon populations	30, 109
DT160	ST19	Wild birds, extended outbreak in New Zealand, multiple hosts	31–33, 35
ND	ST313	Disseminated disease in sub-Saharan Africa, 1960 to present	43, 62
ND	ST213	Emergent clone in Mexico in 2002	39

a *S*. 4,[5],12:i:−.

The epidemiological record of *S*. Typhimurium phage types in domestic animals and humans in England, Wales, and Germany is characterized by successive waves of a dominant phage type, with each wave spanning approximately 10 to 15 years and accounting for over 50% of all isolates at the peak of the epidemic (14). Strains of DT9, DT204, DT104, and the current *S*. 4,[5],12:i:− (monophasic Typhimurium variant) DT193/DT120 have in turn been the dominant strains since the early 1960s in Germany (21). However, the reason for the decline and replacement by successive strains is not well understood. Two characteristics of strains that become most prevalent are multidrug resistance (MDR) and a relatively broad host range (14). For example, the DT104 strain was initially associated with cattle but became widespread in pigs, poultry, and some wild animal species and had a typical pentaresistance pattern of ACSSuT (ampicillin, chloramphenicol, streptomycin, sulfonamides, and tetracycline) (24), and while *S*. 4,[5],12:i:− DT193/DT120 is primarily associated with pigs, the strain has since spread to other species and has the resistance pattern ASSuT (25, 26).

Dominant MDR pandemic clones like DT9, DT204, DT104, and the current *S*. 4,[5],12:i:− (monophasic) DT193/DT120 exemplify the broad-host-range pathogens once considered typical of *S*. Typhimurium. However, many examples of host-adapted *S*. Typhimurium pathovariants have also been described. The host adaptation of these variants is supported by the epidemiological record, and in some cases, descriptions of clinical disease and experimental infections suggest that they are also pathovariants. Some phage types exhibit a restricted distribution to particular zoonotic reservoirs and are typically associated with gastroenteritis in humans (DT8 and U288), while others are exclusively associated with severe disseminated diseases in wild animal host populations but rarely associated with disease in humans (DT2, DT99, and DT56). The host adaptation of *S*. Typhimurium variants was recognized as long ago as 1937, when it was noted that Salmonella from ducks has a distinct biotype (15). *S*. Typhimurium DT8 outbreaks of foodborne disease reported in Germany (15), Ireland (27), and the United Kingdom (28, 29) have been traced back to the consumption of raw or undercooked duck eggs, but this phage type is rarely isolated from other zoonotic reservoirs (13, 15). Despite the considerable degree of host adaptation exhibited by DT8 strains, they retain full virulence in humans. The reported gastrointestinal symptoms of DT8 in humans, i.e., diarrhea and, in most cases, fever and nausea, are similar to those described for other nontyphoidal serovars, and over half of infections are associated with bloody diarrhea (27). *S*. Typhimurium variants of DT56 (DT56var) and DT40 have long been associated with passerine salmonellosis, an often-fatal infection in birds in the order Passeriformes that includes song birds, such as the finch and sparrow (30). These phage types are dominant isolates from the carcasses of finches and sparrows in the United Kingdom but are rarely isolated from livestock and poultry. Despite their adaptation to circulation in avian populations, they are able to cause gastroenteritis in people (31). *S*. Typhimurium DT160 is also a phage type typically associated with the environment and moribund wild birds but not with nonavian species (31–34). However, this phage type was also associated with an extended outbreak in New Zealand between 1998 and 2012, with over 3,000 reported cases of salmonellosis in people. During this time, DT160 was, atypically, widely distributed among poultry, bovines, and felines, as well as in wild birds (35, 36), and may represent a distinct variant.

Some phage types are particularly associated with specific wild bird populations and rarely cause disease in people. Strains of the *S*. Typhimurium variant Copenhagen with phage types DT2 and DT99 are examples, exhibiting exquisite adaption to the pigeon, where they cause severe disseminated disease (30). In Germany, there was considerable concern over the potential risk to public health from *S*. Typhimurium variant Copenhagen (*S*. 4,12:i:1,2) that was common in pigeons, as this variant of Typhimurium was also associated with disease in humans. However, ribotyping, biotyping and phage typing of *S*. Typhimurium variant Copenhagen isolates from human clinical infections and pigeons indicated a distinct variant in the pigeon population (37). Epidemiological data from Germany spanning 25 years indicated that DT2 and DT99 accounted for virtually all pigeon isolates but were rarely isolated from clinical infections in humans (15). DT2 and DT99 strains exhibit differing and atypical pathogenicities in the mouse and bovine ileal loop models of infection. DT2 and DT99 strains are attenuated in experimental infections of mice and have differing abilities to induce fluid accumulation in the bovine ileal loop model of colitis (19, 38).

Other groups of strains with distinct epidemiology for human or animal infection have been defined using MLST. These include *S*. Typhimurium sequence type 213 (ST213), which was reported to account for a large proportion of clinical infections in Mexico, and ST313, associated with disseminated disease in sub-Saharan Africa. *S*. Typhimurium ST213 emerged around the year 2001 in several states in Mexico and accounted for over 50% of isolates by 2005, displacing the previously dominant ST19 strains (39, 40). The ST213 strains were characterized by plasmid-borne MDR, including resistance to extended-spectrum cephalosporins and the lack of the virulence plasmid pSLT. One ST213 strain isolated from a child with disseminated disease was attenuated in experimental infections of genetically susceptible mice, probably due to the lack of the virulence plasmid (41). ST213 strains have not been reported outside Mexico, and the explanation for the rapid expansion in this clonal group is not known.

*S*. Typhimurium ST313 strains accounted for over 90% of bloodstream isolates from a number of countries across sub-Saharan Africa between 1995 and 2010 (42, 43). Disseminated nontyphoidal Salmonella infection (invasive NTS) has been recognized as a common cause of bloodstream infection in sub-Saharan Africa since the late 1980s (5, 44). Disseminated disease is generally uncommon in infections caused by nontyphoidal Salmonella serovars, such as *S*. Typhimurium, and the high incidence of ST313 in sub-Saharan Africa is at least in part associated with the relatively large population of immunocompromised people (5). The major risk factors for disseminated NTS in sub-Saharan Africa are HIV coinfection in adults and malaria or HIV coinfection or malnutrition in children. Depletion of T helper type 17 (T_H_17) cells (45) and dysregulation of the humoral response (46) have been identified as contributing factors in HIV infection. Increased intestinal wall permeability (47) and impaired macrophage (48) and neutrophil (49) function contribute to comorbidity with malaria. The lack of large-scale surveillance of Salmonella in zoonotic reservoirs and people in sub-Saharan Africa limits our understanding of the degree to which the ST313 genotype contributes to the outcome of infection and whether it is truly a host-adapted pathovar. However, in one study in Kenya, isolates to similar that of the index case could not be found in food, the environment, or animals in close contact with the index case (50). Furthermore, despite their prevalence in disseminated disease in sub-Saharan Africa, *S*. Typhimurium ST313 strains are not commonly isolated outside the continent (43). Few strains isolated during routine surveillance in England and Wales were ST313, and most were genetically distinct from the two epidemic ST313 clades prevalent in sub-Saharan Africa (43, 51). However, ST313 strains from England and Wales that were present in the epidemic ST313 clade prevalent in sub-Saharan Africa were significantly more likely to be bloodstream infections (52), suggesting that this particular clonal group of ST313 may be innately predisposed to cause disseminated disease. Experimental infections of mice and primates largely support the view that ST313 strains have altered pathogenicity compared with those of commonly used laboratory strains, such as strains SL1344 and ATCC 14028. An ST313 isolate hyperdisseminated from the intestine to systemic sites in the murine model of infection and in poultry (16, 17, 53). Furthermore, ST313 strains exhibit decreases in intestinal pathology in mice, fluid accumulation and migration of inflammatory cells to the intestinal lumen in bovine ileal loops, and diarrhea in rhesus macaques (53, 54).

## POPULATION GENETICS OF *S*. TYPHIMURIUM PATHOVARIANTS

**FIG 2:**
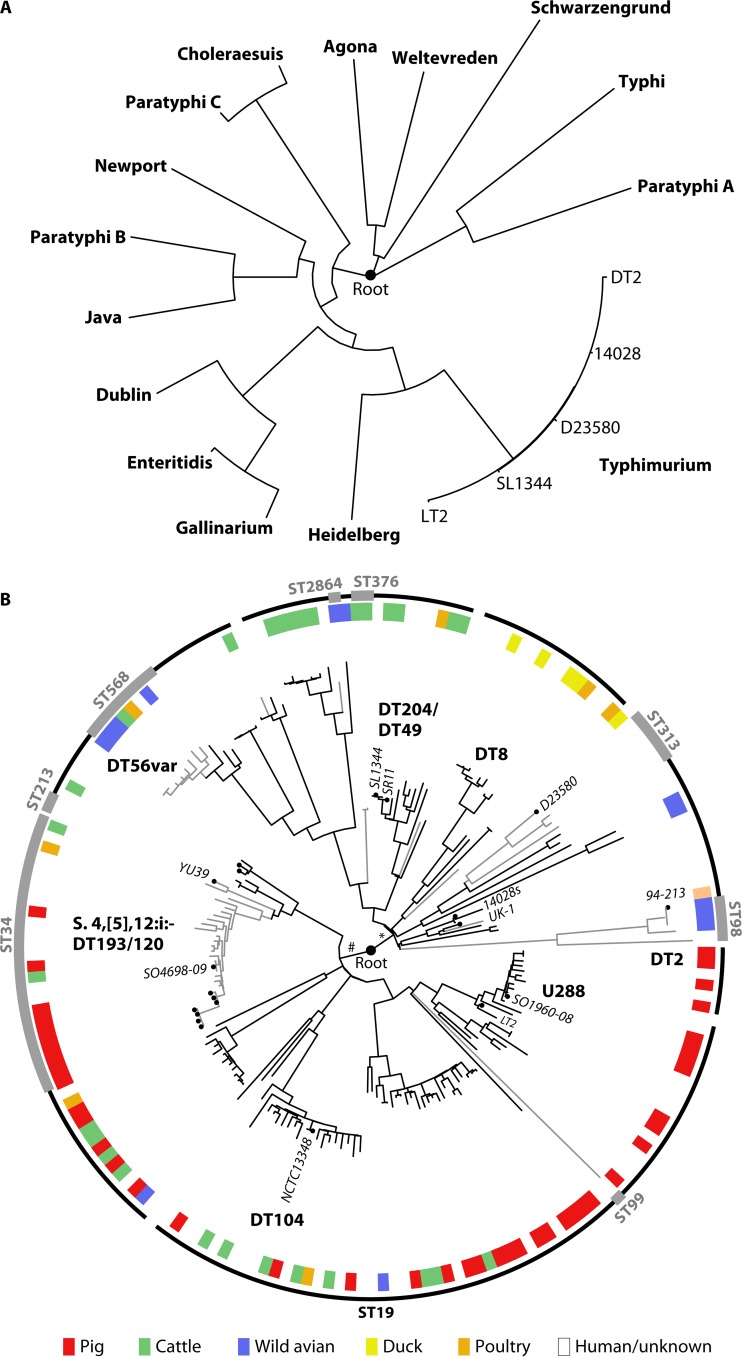
Phylogenetic relationships of Salmonella enterica subspecies I serovars and *S*. Typhimurium strains. Maximum-likelihood trees of 15 S. enterica subspecies I serovars (A) and 194 *S*. Typhimurium strains (B) based on sequence variation in the core genome. Trees are midpoint rooted (filled black circle). (B) The inner circle indicates the source animal host species for each *S*. Typhimurium strain, with color code as shown in the key. The outer circle indicates ST19 (black line), other STs (gray box), and strains with an unassigned ST (line break). Strains with a published complete, closed genome sequence are indicated by small black circles at leaf nodes, and the names of key strains are in italics. The locations of key clades are indicated with boldface black font. The positions of two major clades containing strains with distinct epidemiology are indicated near the root (# and *).

Whole-genome sequence analysis has brought new insights to Salmonella taxonomy. The phylogenetic tree of S. enterica subspecies I revealed that serovars are located on deeply rooted branches in a star-shaped topology (Fig. 2A). The length of each branch is proportional to the number of single nucleotide polymorphisms (SNPs) and represents the level of divergence. Branches closer to the ancestor (root) are defined by SNPs that are shared by multiple strains, while terminal branches are defined by strain-specific SNPs. *S*. Enteritidis and *S*. Gallinarum are relatively closely related, yet they exhibit profound differences in host range and pathogenicity, being broad host range and highly host adapted to Galliformes birds, respectively, highlighting the potential for evolution in relatively short periods of time (55, 56). *S*. Typhimurium is on a deeply rooted branch, distinct from other serovars, with diversification at the extreme terminus of the lineage.

Historically, collections of strains intended to represent *S*. Typhimurium's genetic diversity in natural populations have been established based on phage type (57) and multilocus enzyme electrophoresis (58). The whole-genome sequences of some of the strains from these collections, along with additional contemporary strains representing the common phage types isolated from animals and human clinical cases, have been used to contextualize epidemics in sub-Saharan Africa (43) and the United Kingdom (59). The phylogeny of *S*. Typhimurium reveals a star-shaped tree topology, similar to that for serovars of S. enterica subspecies I but on a considerably smaller scale of sequence divergence (Fig. 2B). Some lineages within the *S*. Typhimurium tree have multiple short terminal branches, characteristic of clonal expansion of epidemic clones (Fig. 2B). The diversity within *S*. Typhimurium based on the number of SNPs in the core genome is approximately 2 orders of magnitude lower than that within S. enterica subspecies I. *S*. Typhimurium has accumulated around 20,000 to 40,000 SNPs since divergence from a common ancestor shared with other S. enterica serovars, corresponding to an average nucleotide identity (ANI) of 98 to 99% (60, 61). In contrast, *S*. Typhimurium strains accumulated approximately 400 to 600 SNPs, corresponding to approximately 99.99% ANI (19, 59, 62). For comparison, Salmonella genomes exhibit around 80% ANI with those of its nearest neighbor, Escherichia coli (63).

Two high-order subclades are evident in the *S*. Typhimurium tree; although only weakly supported by sequence variation data, they nonetheless predominantly contain *S*. Typhimurium strains with distinct epidemiology. The first (Fig. 2B, pound sign [#]) contains strains almost entirely from cattle, pigs, and poultry with considerable diversity at the terminal branches, formed by clonal expansion of the most recent pandemic clones, the DT104 clade and the monophasic *S*. Typhimurium DT193/DT120 clade (59, 64). A clade in which all animal-derived isolates were from pigs included many strains of phage type U288 that are epidemic in UK pig herds, suggesting that this could be a pig-adapted clade (65). The second major subclade (Fig. 2B, asterisk [*]) is characterized by lineages that are deeply rooted, with less evidence of clonal expansion in the sampled isolates, and contains strains isolated from a considerably greater diversity of host species. Although strains from cattle and poultry are represented, no pig isolates are present, but instead, strains from wild birds, including passerines and pigeons, are present in multiple lineages. The prototypical laboratory strain SL1344 is present, along with *sopE*-bearing strains of phage types DT204 and DT49 (66) that are likely related to strains that were epidemic during the 1970s in Europe (14, 21). The second major subclade also contains host-adapted pathovariants, including two lineages containing DT2 and DT99 strains that are host adapted to pigeons (19), a lineage containing DT56 strains associated with passerine birds (67), the ST313 lineage containing two epidemic expansions associated with disseminated nontyphoidal salmonellosis in sub-Saharan Africa (43), and a lineage including DT8 strains adapted to circulation in duck populations (29).

The diversity of sequence type (ST) within the *S*. Typhimurium clade is low and, in most cases, does not discriminate between subclades on major lineages. The vast majority of isolates are ST19, reflecting the ancestral type; these account for most of the strains that are associated with gastroenteritis and exhibit considerable diversity (Fig. 2B). A few additional STs are present as discrete subclades, and in some cases, these have undergone considerable divergence and establishment of multiple lineages, as is the case for ST313 (43).

## COMPARATIVE GENOMICS OF BROAD-HOST-RANGE *SALMONELLA* TYPHIMURIUM

The vast majority of what is known of the physiology, biochemistry, genetics, and pathogenicity of Salmonella comes from over 70 years of experimental research using just three strains of *S*. Typhimurium: LT2, SL1344, and ATCC 14028. Two additional strains, SR11 and UK1, have been used by some laboratories, but these are closely related to SL1344 and ATCC 14028, respectively (Fig. 2B) (68). *S*. Typhimurium LT2 strain 85 was originally isolated at the Stoke-Mandeville Hospital in the United Kingdom in 1946 (57), and following its use in the first transduction experiments (69), it became the strain of choice for many investigations (70). However, the utility of the LT2 strain for studying host-pathogen interactions was limited by its attenuation in the murine typhoid model, at least in part due to defective RpoS (71). Consequently, strains SL1344 and ATCC 14028, isolated from bovine and chicken hosts, respectively, are commonly used in laboratories for pathogenesis studies (72, 73). Both SL1344 and ATCC 14028 exhibit virulence in a wide range of hosts, including mice, cattle, and poultry, with a notable difference being that SL1344 bears the *sopE* gene, located on a lysogenic phage, that results in increased enteropathogenesis (74). The evidence from epidemiology and experimental infection of animals would suggest that SL1344 and ATCC 14028 represent the typical broad-host-range phenotype classically associated with *S*. Typhimurium. However, it is noteworthy that SL1344 and ATCC 14028 are present in the part of the *S*. Typhimurium phylogenetic tree that is distinct from recent MDR pandemic clades (DT104 and *S*. 4,[5],12:i:− DT193/DT120) and more closely related to lineages containing pathovariants adapted to avian hosts (Fig. 2B). The divergence of SL1344 and ATCC 14028 highlights the need to understand the impact of genotypic differences on pathogen-host and pathogen-environment interactions in order to interpret the large body of work with these strains with regard to disease caused by currently circulating pandemic strains.

The sizes of the chromosomes of LT2, SL1344, and ATCC 14028 are similar, differing by no more than about 20 kb (LT2, 4,857,432 bp; SL1344 4,878,012, bp; and ATCC 14028, 4,870,265 bp) (60, 72, 73). The genomes of the three strains are highly syntenic and show conservation of Salmonella pathogenicity islands (SPIs) (75), and most of the variation in coding capacity is restricted to prophage sequences and plasmids. This was also true for a larger collection of 20 complete *S*. Typhimurium genomes (76). The gene model for the chromosome of strain SL1344 has 148 genes not present in strain LT2, and these are almost entirely accounted for by the presence of partial and complete prophage elements SLP281, SLP203, and SLP289 that are absent from LT2 and regions of variable sequence in the FELS-2 prophage (73). The LT2 genome likewise contains genes absent from SL1344 due to additional prophages.

The variable presence of plasmids also contributes significantly to differences in coding capacity. All three laboratory strains contain the 90-kb virulence plasmid pSLT (77), while strain SL1344 carries an additional 112 genes on two plasmids, pRSF1010 and pCol1b9 (73). The initial annotation of strain LT2 resulted in a gene model consisting of 4,489 genes on the chromosome (60). However, improved gene prediction models and mapping of transcription start sites to the SL1344 whole-genome sequence assembly using RNA sequence data resulted in 4,899 predicted genes (73).

The coding capacity of bacterial genomes can be considerably decreased by the formation of pseudogenes, due to sequence polymorphisms, insertions, or deletions introducing a premature stop codon, which is termed “genome degradation” (78). Host-restricted serovars like *S*. Gallinarum and *S*. Typhi contain around 200 to 300 pseudogenes, up to 5% of the original coding capacity (55, 61). Overrepresented degradation of genes associated with intestinal colonization and persistence, including those involved in anaerobic metabolism, is consistent with the loss of functions no longer required for the disseminated-disease lifestyle adopted by these serovars (79). *S*. Typhimurium strain SL1344 contains 54 pseudogenes, 38 of which are also pseudogenes in LT2, and a further 6 are also pseudogenes in strain ATCC 14028 (60, 72, 73). Just 10 pseudogenes are specific to strain SL1344, and a similar number are specific to ATCC 14028, with reference to these three strains (72). Hence, most pseudogenes in commonly used laboratory strains formed in a common ancestor of *S*. Typhimurium and so could not contribute to strain-specific characteristics. Strain-specific pseudogenes are of potential interest, as they could contribute to strain-specific phenotypes. In SL1344 and ATCC 14028, strain-specific pseudogenes generally encode proteins of unknown function or of a nonspecific nature but, notably, they do not include virulence genes or regulators.

Care must be taken in the interpretation of the potential impact of pseudogene formation, as some proteins may remain functional due to a small truncation or if proteins are composed of multiple domains. Even a relatively large truncation that nonetheless results in retention of functional domains may not abrogate function entirely. For example, the RatB protein is present in the full-length form of 2,435 residues in strain LT2 (RatB^LT2^), but the introduction of a frameshift in the coding sequence in both ATCC 14028 and SL1344 results in a protein of 1,947 residues. However, the truncated RatB of ATCC 14028 (RatB^ATCC 14028^) appears to remain functional, since deletion of the *ratB* gene resulted in decreased colonization of the cecum in experimentally infected mice, a phenotype that was complemented by the 1,947-residue-encoding *ratB* (80). A possible explanation for this observation is that RatB is composed of repeating sequence modules, and loss of a subset of these does not result in loss of function. The primary amino acid sequence of RatB^LT2^ contains eight imperfect repeats, and that of RatB^ATCC 14028^ contains six complete repeats. The assumption that pseudogene formation is equivalent to a null mutation in all cases may therefore result in misleading conclusions. In practice, the exhaustive functional analysis of all polymorphisms that lead to pseudogenes is impractical in comparative genomics studies, and this remains an important caveat to be considered.

Complete, closed, whole-genome sequences have been determined for the MDR pandemic strains *S*. Typhimurium DT104 (strain NCTC13348) and *S*. 4,[5],12:i:− DT193/DT120 (strain SO4698-09) and reveal extensive similarity to model laboratory strains, with some key differences that are likely to have contributed to their success (59, 64, 81). A key feature of DT104 is the presence of Salmonella genomic island 1 (SGI-1), an integrative conjugative element bearing a number of genes conferring resistance to multiple drugs that can be mobilized with a helper conjugative plasmid (82). *S*. 4,[5],12:i:− DT193/DT120 strain SO4698-09 contains a composite transposon inserted adjacent to the *fljB* and *iro* loci that encodes resistance to multiple antibiotics and mercury. The strain additionally contains a putative integrative conjugative element (ICE), termed SGI-4, that was acquired shortly before clonal expansion and is absent from all other Salmonella genomes reported to date. It bears genes predicted to confer resistance to arsenic, copper, and silver. The presence of SGI-4 correlated with enhanced resistance to copper, a phenotype that has been proposed have possibly contributed to the success of the *S*. 4,[5],12:i:− DT193/DT120 clone in pig herds, where copper is used as a growth promoter due to its antimicrobial properties (59). Complete, closed genome sequences are also available for a number of other *S*. Typhimurium strains (Fig. 1B), but a detailed comparative genome analysis has not been reported. These include whole-genome sequences of 10 *S*. Typhimurium strains from cattle, beef products, and human clinical cases in the United States, including several *S*. 4,[5],12:i:− isolates similar to the epidemic clade in the United Kingdom (83).

## COMPARATIVE GENOMICS OF HOST-ADAPTED *S*. TYPHIMURIUM PATHOVARIANTS

Strains of *S*. Typhimurium ST313 form a clade mainly associated with invasive nontyphoidal Salmonella disease in sub-Saharan Africa. The complete and closed whole-genome sequence of strain D23580 revealed a genotype distinct from those of broad-host-range strains commonly used in the laboratory (62). It exhibits considerably more variation in gene content and coding capacity than laboratory strains like SL1344, LT2, and ATCC 14028. D23580-specific genes are mainly due to the presence of two novel prophages, BTP1 and BTP5, and two apparently cryptic plasmids, pBT1 and pBT2 (62). Strain D23580 also carries at least 25 additional genes on its virulence plasmid pSLT-BT, due to the presence of a composite Tn*21*-like transposon, including antimicrobial and mercury resistance genes. Outside these regions, the chromosome of D23580 is around 15 kb shorter than those of laboratory strains like SL1344 due to four deletions impacting the coding sequences of 20 genes. Some of the deletions affect virulence genes, a functional class unaffected by genome degradation, in laboratory strains like SL1344 and 14028; examples include the *pipD* gene borne on SPI-5, involved in enteropathogenesis and intestinal persistence (84, 85), *mig-13* and *pagO*, involved in persistent colonization in mice (85), and the *allP* and *allB* genes, involved in allantoin metabolism (86). Deletions on a similar scale in other *S*. Typhimurium strains have not been reported to date; indeed, with the exception of mobile elements, such as insertion sequence (IS) elements, ICEs, and prophages, deletions of more than a few bases occurring in strains of *S*. Typhimurium appear to be rare. Strain D23580 also contained a greater number of strain-specific pseudogenes than SL1344 and 14028. With reference to the pseudogene content of strain LT2, a total of 24 pseudogenes specific to strain D23580 were reported (62, 87). Notably, 11 were also pseudogenes or absent from the *S*. Typhi or *S*. Paratyphi A genomes, perhaps the result of convergent evolution to a disseminated disease lifestyle. These degraded genes include regulators and a number of genes of unknown function. Several pseudogenes in D23580 have assigned functions, including *ratB*, involved in intestinal persistence, *lpxO*, involved in modification of the lipid A component of the membrane, *sseI*, encoding an effector protein secreted by the SPI-2-encoded type III secretion system, and *bcsG*, involved in cellulose biosynthesis (16, 62, 80, 87). Two, *bcsG* and *sseI*, have been linked to ST313-specific phenotypes in the environment and the host. The *bcsG* loss-of-function mutation was linked to loss of multicellular behavior and *sseI* to hyperdissemination from the intestine to systemic sites in the murine model of infection (16, 87). Finally, a single nucleotide polymorphism in the promoter region of the *pgtE* gene results in increased expression of the virulence factor PgtE, increasing resistance to serum killing and modulating virulence in chicken infections (18).

A complete, closed reference genome is also available for strain 94-213, a representative of *S*. Typhimurium DT2, associated with severe disseminated disease in pigeons (19). Remarkably, despite the host adaptation exhibited by this pathovariant, which is supported by epidemiological data (15, 30) and experimental infections of mice (19), calves (38), and chicks (19), the gene content outside phage and IS elements is identical to that of strain SL1344 (19). However, considerable genome degradation was present in the DT2 genome, due to 21 pseudogenes that were full length in strains LT2, SL1344, DT104, and D23580. Inactivation of two of these genes, *pcgL* and *gtgE*, has previously been implicated in hyperdissemination and host restriction, respectively (88, 89). PcgL is a periplasmic d-alanyl–d-alanine dipeptidase whose inactivation results in hypervirulence in mice by an as-yet-undefined mechanism and represents an example of loss of antivirulence genes (90). GtgE is a cysteine protease effector protein that targets a family of Rab-GTPases and is widely distributed in broad-host-range Salmonella strains (unpublished observations). Its absence in *S*. Typhi has been proposed to determine, at least in part, the host restriction of this serovar (89) and may therefore also contribute to the epidemiology of DT2. Inactivation in strain SL1344 of genes orthologous to pseudogenes in DT2 did not result in attenuation of virulence in mice, suggesting that combinations of these mutations may be required for the phenotype (19). However, DT2 was found to have a modified transcriptional response during culture at elevated temperature, mimicking a feature of the avian host (19). A number of genes were expressed at decreased levels in DT2 compared to their expression in the broad-host-range strain SL1344, including many genes involved in motility, flagellum biosynthesis, and chemotaxis. Culture of DT2 at an elevated temperature also resulted in a decreased inflammatory response upon interaction with epithelial cells *in vitro*, consistent with the disease pathology described for this pathovariant. *S*. Typhi also downregulates flagella in the host, resulting in a decreased inflammatory response. This results from regulation by the TviA regulator, which was acquired by *S*. Typhi on Salmonella pathogenicity island 7 (SPI-7) (91). However, DT2 does not encode additional regulators, and the genetic basis of the DT2-specific transcriptional response to elevated temperature is unknown (19).

## MOLECULAR EPIDEMIOLOGY, PHYLOGEOGRAPHY, AND MICROEVOLUTION OF *SALMONELLA* TYPHIMURIUM DURING CLONAL EXPANSION

Next-generation-sequencing technologies have made it possible to determine the whole-genome sequences of tens of thousands of bacterial pathogens, enabling discrimination of isolates at the highest possible resolution and the observation of short-term evolution (92). Variations in genomes arising from single-nucleotide polymorphisms (SNPs) and insertions and deletions are readily identifiable. Sequence variation, along with spatiotemporal data of the strain, has been used to infer the spread of epidemic clones nationally and globally, as well as for outbreak detection, source attribution, computation of molecular clock rates, and reconstruction of maximum-likelihood and time-dependent phylogenetic trees using Bayesian inference. Assembled sequences have been used to determine the pangenome, which has shed light on the gene flux that contributes to macro- and microevolutionary mechanisms, including host adaptation and antimicrobial resistance (93). Draft genomes can be assembled with short read sequences from next-generation technology that, while typically composed of 50 to 150 contiguous sequences (contigs), allow the core and accessory genomes of bacterial populations to be determined (94, 95).

Estimates of long-term substitution rates in bacteria, calibrated with geological and ecological events, suggest that Salmonella and E. coli shared a common ancestor 100 to 150 million years ago, corresponding to a clock rate of around 10^−10^ to 10^−9^ substitutions per site per year (96). Based on this calculation, we would not expect to observe changes in the genome sequence associated with clonal expansion spanning less than about 100 years. The sequence of an *S*. Paratyphi C genome recovered from an 800-year-old human skeleton revealed the somewhat faster clock rate of 9.4 × 10^−8^ substitutions per site per year (97). Comparable substitution rates for the *S*. Typhimurium clade as a whole have not been determined, as there was a lack of correlation between the accumulation of SNPs (branch lengths) and dates of isolation (68). However, several studies have reported short-term (10 to 50 years) substitution rates of *S*. Typhimurium during clonal expansion in epidemics or outbreaks. A substitution rate of 1.2 × 10^−6^ per site per year was calculated during clonal expansion of a DT135 clone on a poultry farm in Australia over about 14 years, corresponding to 5 SNPs per genome per year (68). A lower rate of approximately 3 × 10^−7^ per site per year, or 1 or 2 SNPs per genome per year, was reported for two ST313 epidemic clones in sub-Saharan Africa over a 45-year period (43), a global collection of DT104 isolates (98), and a DT160 epidemic clone over a 14-year period in New Zealand (35). Molecular clock rates have been used to estimate the dates of major branching in phylogenetic trees of epidemic clades and, in combination with site-of-isolation data, the spatiotemporal spread of clones. In the DT160 epidemic in New Zealand (36), ancestral date reconstruction using whole-genome sequences of 109 isolates predicted a shared common ancestor around August 1997 (35). Furthermore, strains isolated from human clinical infections, poultry, wild birds, felines, bovines, equines, canines, and the environment were dispersed throughout the phylogenetic tree. These analyses were used to infer a point source of the epidemic following introduction to New Zealand around 1997, followed by rapid spread through many animal populations (35). Similar Bayesian approaches estimated the emergence of the DT104 pandemic clade as an antibiotic-sensitive clone around 1948 and the acquisition of the MDR phenotype around 10 years later by a single sublineage, which later underwent clonal expansion and considerable diversification during the pandemic of the 1990s (98).

The vast majority of disseminated *S*. Typhimurium infections in sub-Saharan Africa between 1995 and 2010 resulted from strains from two lineages (I and II) of ST313, (42, 43). Each lineage contains a distinct epidemic strain that initiated clonal expansion around 1965 and 1985, respectively. The common ancestor of lineages I and II existed some time before the clonal expansion of either strain, around the year 1800 (52). Clonal expansion was concomitant with the independent acquisition by each epidemic strain's ancestor of distinct composite transposons bearing genes conferring resistance to front-line drugs used to treat disseminated Salmonella infections in sub-Saharan Africa. Epidemiological evidence from Malawi supports the inference from sequence analysis that the lineage I clone emerged first and was replaced rapidly in a clonal sweep by the lineage II strains (99). The phylogenetic relationship of ST313 isolates from countries across the region contained a strong geographical signature, with isolates from each country clustering together. The inferred spatiotemporal spread of the ST313 epidemic was similar to that of the spread of HIV across sub-Saharan Africa, emerging initially in central Africa around 1980 and then spreading east and subsequently south, before finally arriving in West Africa some years later (43). ST313 strains in human infections are rare outside sub-Saharan Africa. In routine surveillance in the United Kingdom, just 2.6% of *S*. Typhimurium isolates from human clinical cases of Salmonellosis were ST313. However, most of these were not part of the lineage I or II epidemic groups from sub-Saharan Africa, but rather, were on closely related but distinct lineages, representing previously unknown genetic diversity within ST313. Remarkably, of the few UK isolates that were part of the Africa-associated lineage II, the majority were associated with travel to sub-Saharan Africa, and many were associated with extraintestinal infections. ST313 strains from the UK that were associated with disseminated disease but not present in the lineage II clade were extremely rare (52). The paucity of epidemiological surveillance across sub-Saharan Africa precludes traditional approaches to investigate epidemics, but the example of ST313, for which whole-genome sequences from a relatively small number of isolates were employed to reconstruct the phylogenetic history of isolates, demonstrates the utility of these approaches in resource-poor settings.

Nucleotide substitutions that accumulate in genes are either synonymous (conserving the primary amino acid sequence) or nonsynonymous. The comparative rate of synonymous to nonsynonymous SNPs (*dN*/*dS*) has been reported to be approximately 0.5 in the branches of a tree defining reference strains of *S*. Typhimurium (62), and this was also true over short time scales within epidemic clades (43, 68). This has been interpreted as evidence that around half of all nonsynonymous substitutions are deleterious and rapidly removed from the population (68). Most of the remaining nonsynonymous SNPs are assumed to have little impact on the function of the encoded protein or, alternatively, to result in changes in the function of the protein that are either not under selective pressure or, indeed, are beneficial. One scenario in which mutations may be beneficial is in the evolution of host-adapted pathovariants. In this case, genes required for circulation in multiple hosts are no longer required, since the pathovariant is restricted to a single host species. These may include genes encoding adhesins that recognize species-specific receptors and those involved in nutrient scavenging and associated metabolic genes no longer required in the restricted host (79). A profile hidden Markov model (HMM)-based method to identify functional divergence in orthologous proteins called delta-bit score (DBS) (100) has been used to prioritize polymorphic proteins in the *S*. Typhimurium DT2 pathovariant for further investigation of their role in phenotypes of interest (19). Polymorphic proteins that return a lower bit score than the orthologue from a broad-host-range comparator strain contain changes in conserved domains that are therefore more likely to have altered function. The frequency distribution of DBS values for DT2 compared to those of SL1344 orthologues revealed a skew from a normal distribution toward a positive score (19), similar to that observed in a comparison of the host-adapted serovar *S*. Gallinarum to the closely related broad-host-range serovar *S*. Enteritidis (100). This methodology led to the identification of a polymorphism in the *tar* gene of DT2 that altered the chemotactic response in the DT2 pathovar. Flagellum-mediated motility and chemotaxis are known to be important during the intestinal phase of infection (101, 102), and the *tar* polymorphism is consistent with evolution of DT2 from an intestinal to an extraintestinal pathovar.

Considering the generally limited differences in gene content when comparing the genomes of pathovariants of *S*. Typhimurium on distinct lineages, a considerable amount of microevolution occurs over short periods of time during clonal expansion of epidemic clones. This variation has the potential to alter susceptibility to antimicrobials, antigenicity, metabolism, and the outcome of infection. Although the MDR phenotype of epidemic clones of *S*. Typhimurium appears to be important for their success, considerable sequence variation is observed in antimicrobial resistance (AMR) gene loci during clonal expansion. Variation in SGI-1 of the *S*. Typhimurium DT104 strains is known to occur through recombination events (82), but analysis of the genome sequences of 359 strains isolated over a 22-year period revealed frequent and recurrent loss or gain of genes throughout the clade, ranging from the entire SGI-1 to single AMR genes (64). Similar observations were made for the AMR genes in the *S*. 4,[5],12:i:− DT193/DT120 epidemic (59). Additional genotypic variation outside the AMR locus was observed in the genome sequence of the *S*. 4,[5],12:i:− DT193/DT120 epidemic clone due to gene flux from complex deletions around the *fljB* locus and the acquisition of prophages, an integrative conjugative element, and plasmids (59). The genome flanking the *fljB* is hypervariable within *S*. 4,[5],12:i:− DT193/DT120 epidemic strains. Reconstruction of the ancestral state with respect to this locus indicated that early in the *S*. 4,[5],12:i:− epidemic, the *fljB* locus was intact and the strains were biphasic. The presence of a composite transposon adjacent to the *fljB* locus that contained IS6 sequences known to mediate local deletions appears to have resulted in multiple deletion events during clonal expansion. The deletions resulted in the emergence of the monophasic phenotype within the clade on multiple occasions, due to deletion not only of the *fljB* locus but also up to 36 kb affecting 33 additional genes (59). The *S*. 4,[5],12:i:− clade also acquired on multiple independent occasions a novel phage designated mTmV that carried the *sopE* gene. SopE is an effector protein with guanine exchange factor activity for host Rho GTPases, which are involved in invasion of epithelial cells and induction of host proinflammatory response (103). It has been associated with epidemic clones in the past (104). It impacts pathogenicity (103) and imparts a competitive advantage to Salmonella bacteria competing with host gut microbiota (74), and therefore, its acquisition is likely to have had a considerable impact on the epidemic. Indeed, its acquisition resulted in clonal expansion of strains bearing *sopE* within the epidemic, resulting in an increase in their prevalence from low levels in 2005 to a third of isolates carrying the *sopE* gene by 2010 in the UK epidemic (59). The transfer of phage in the intestine, boosted by the inflammatory response of the host, has been reported for the sopEΦ phage from SL1344 in experimental infections of mice, demonstrating the potential for microevolution that changes the nature of epidemics (105).

## CONCLUSIONS

The combination of complete, closed, whole-genome sequences of key reference strains of *S*. Typhimurium and thousands of draft whole-genome sequences representing a considerable amount of diversity within the group is dramatically changing our understanding of this prototypical Salmonella serovar. A vast body of literature describes the biochemistry, physiology, and host-pathogen interactions of a vanishingly small number of strains of *S*. Typhimurium. For the purposes of quantitative risk assessment for foodborne disease, Salmonella has been considered homogenous, but a greater understanding of the diversity of genotypes and their interactions with the host and environment now has the potential to refine how we view the risk and provide the tools needed to design appropriate interventions. The genomic variation of *S*. Typhimurium pathovariants provides a window into the events that lead to the formation of new pathogens. The gene flux, genome degradation, and alteration of transcriptional responses associated with adaptation to new hosts and environmental niches are relatively recent adaptations in *S*. Typhimurium pathovariants. Perhaps the most exciting insights from high-throughput sequencing are applications in surveillance and molecular epidemiology that provide insights into source attribution and the dynamics of epidemic spread, impossible from traditional surveillance approaches. The globalized food chain requires global solutions to control foodborne pathogens, and the combination of technological and bioinformatic developments will make it possible to integrate pathogen surveillance around the world.
